# Case of Scirrus of the Stomach, of Twenty Years’ Standing

**Published:** 1853-11

**Authors:** N. E. Ballou

**Affiliations:** Carlton Centre, N. Y.


ART. II.—*Case of Scirrus of the Stomach, of twenty years' standing. Perforation. Peritonitis and Death. Autopsy.* By N. E. BALLOU, M.D., Carlton Centre, N. Y.

Believing that the following case possesses some interest to the profession, from its presenting features analogous to dyspepsia, with which, no doubt, scirrus is often confounded, I am induced to give briefly an account of some of the prominent symptoms—the cause of development in this individual case, having previously a scirrous diathesis:*

D. V. S., æt. 38; bilious sanguine temperament; married; occupation, drover; had, during twenty years, suffered frequently from paroxysms of "pain of the stomach" (as he usually termed it) followed in a longer or shorter period by vomiting. For the sake of brevity, and in order to bestow a better arrangement upon what I have to say in attempting to chronicle this, to me, interesting case, I shall divide the period of disease into two epochs, the first embracing fifteen years, and the last five years.

*The cause of development.* When about 18 years of age, the subject of disease while lifting a heavy weight, felt, immediately after the act, a severe pain of the stomach corresponding to the locality of the lesser cul-de-sac. It would not be an anomaly, if at that time a rupture of some of the fibres

---

* An aunt of the deceased died of cancer of the breast, and I have also learned of others of the maternal ancestors who have died of cancerous affections.

of the muscular investment, near the pylorus, had taken place, that ever after was the cause of pain and all of those distressing symptoms which followed through life and matured the diseased condition of the stomach. From that period, which I date as the development of the scirrous affection, onward during fifteen years, nothing worthy of note transpires in the case, only that pain and vomiting were the predominant symptoms, notwithstanding which, the patient at times enjoyed a tolerable measure of health, because, no doubt, of the choice of avocation, *i. e.* cattle-buying or droving, and the horseback exercise connected therewith. The usual symptoms taken together, and denominated dyspepsia, sometimes aggravated, sometimes in a mild form, would describe the leading features of the first epoch.

*The second epoch.* The period which has been denominated the last period, shadowed forth symptoms of a graver character. Instead of the usual symptoms of dyspepsia, excessive pain of the hypochondriac region, constipation, sallowness of the countenance, indicating hepatic disease; vomiting many times the entire ingesta before the food had become changed by the assimilative process; hawking, as if to raise some foreign substance not connected with the organism, and spitting, were symptoms so constant as to be pressingly disagreeable; a superabundance of sputa, so much so that a meal could not be eaten without interruption to give vent to its constant accumulation.

At this extremity of the case the healing art was invoked, to ascertain, if possible, the *fons et origo mali.* Allopathy, homœopathy, and hydropathy, were alike sought, but alike disappointed in their promise of relief.

Opium, the last physician who could even palliate without a promise of a cure, came most opportunely to mitigate, gave "aid and comfort," while the enemy was waging a ruthless war within. Thus progressed a disease as yet occult; hidden from the eye or understanding; invisible; secret; unknown; save by some analogies, in this individual case, none ever had diagnosed scirrus. Pain; vomiting; constipation; emaciation; hawking; rejecting of the food by the stomach without being changed by the assimilative function, were constant symptoms. During all of the time, until a few hours before the fatal termination, there was no pain on pressure, no tenderness, in short no external manifestations of disease, mediate or immediate.

In the spring of 1852, through the intercession of friends, the invalid was induced to try the efficacy of the waters of Avon Springs, as almost the last ray of hope offered in the category of human means; a little time spent there proved that no relief could be had from that source. Hydropathy, in its general principles, now was the only thing untried, the rigid application of which only brought with it the last disappointment. On the 10th of November, at M., the patient was seized with a lancinating pain in the right hypochondrium, which continued in spite of all means. Laudanum was given by the ounce, chloroform applied externally, but all had no effect. Tenderness ensued down the right side of the abdomen, and as the vital energies gradually succumbed to rapidly spreading peritoneal inflammation, the patient sunk at 8 o'clock, P. M. My diagnosis at this time was perforation of the stomach from ulceration, or some other cause.

Autopsy, thirty-six hours after death.

Assisted by Dr. Randall, we made a section of the abdomen, which contained a fourth of a gallon of sero-purulent fluid. Peritoneum highly vascular and injected, exhibiting traces of a severe grade of peritoneal inflammation. Kidneys healthy. Stomach enlarged, but not thickened. Perforation beneath and near the pylorus the size of a five cent piece. Pylorus nearly closed, only admitting the end of the little finger, surrounding which was a scirrous mass the size of a man's fist. The liver was entirely normal in size, appearance, &c. The fluid found in the abdominal cavity, in all probability, was in part increased by escaping through the perforation of the stomach.

